# Illness perception, health literacy, self-efficacy, adherence and quality of life in patients with intermittent claudication – a longitudinal cohort study

**DOI:** 10.1186/s12912-023-01329-2

**Published:** 2023-05-17

**Authors:** Rebecka Striberger, Moncef Zarrouk, Christine Kumlien, Malin Axelsson

**Affiliations:** 1 Department of Care Science, Faculty of Health and Society, Malmö University, Malmö, SE-20506 Sweden; 2 Vascular Centre, Department of Cardiothoracic and Vascular Surgery, Skåne University Hospital, Malmö, Sweden; 3 Department of Clinical Sciences, Malmö, Lund University, Malmö, Sweden

**Keywords:** Illness perception, Self-efficacy, Health literacy, Adherence to treatment, Quality of life, Intermittent claudication, Secondary prevention

## Abstract

**Background:**

Patients with intermittent claudication need lifelong treatment with secondary prevention to prevent cardiovascular events and progression of atherosclerotic disease. Illness perception, health literacy, self-efficacy, adherence to medication treatment, and quality of life are factors influencing patients’ self-management. Knowledge of these factors could be important when planning for secondary prevention in patients with intermittent claudication.

**Aim:**

to compare illness perception, health literacy, self-efficacy, adherence to treatment, and quality of life in in patients with intermittent claudication.

**Methods:**

A longitudinal cohort study was conducted with 128 participants recruited from vascular units in southern Sweden. Data were collected through medical records and questionnaires regarding illness perception, health literacy, self-efficacy, adherence to treatment, and quality of life.

**Results:**

In the subscales in illness perception, patients with sufficient health literacy reported less consequences and lower emotional representations of the intermittent claudication. They also reported higher self-efficacy and higher quality of life than patients with insufficient health literacy. In comparison between men and women in illness perception, women reported higher illness coherence and emotional representations associated with intermittent claudication compared to men. A multiple regression showed that both consequences and adherence were negative predictors of quality of life. When examining changes over time, a significant increase in quality of life was seen between baseline and 12 months, but there were no significant differences in self-efficacy..

**Conclusion:**

Illness perception differs in relation to level of health literacy and between men and women. Further, the level of health literacy seems to be of importance for patients’ self-efficacy and quality of life. This illuminates the need for new strategies for improving health literacy, illness perception, and self-efficacy over time. For example, more tailored information regarding secondary prevention could be provided to strengthen self-management to further improve quality of life in patients with intermittent claudication.

## Background

Patients with intermittent claudication (IC) need lifelong treatment with secondary prevention to prevent cardiovascular events and progression of the atherosclerotic disease [1]. IC is further associated with decreased quality of life (QoL) due to presence of pain and loss of walking ability, which means that living with IC involves managing symptoms to maintain QoL [2]. Importantly, many of these patients do not get secondary prevention support according to the recommendations in current guidelines [1, 3, 4]. Studies have shown that IC is largely unrecognized and under-treated compared to other cardiovascular diseases and that there is a lack of public and patient awareness [5, 6]. The way patients perceive illness is one relevant aspect that determines their health-management behaviour, [7] and it can be improved by patient education [8–10]. Therefore, factors that influence illness perception and QoL could provide important knowledge about how to improve secondary prevention treatment in patients with IC.

Approximately 236 million people globally live with peripheral arterial disease (PAD), [11] where IC is the mildest form, affecting10–20% [12]. IC is characterized by ischemic muscle pain during activity, [1] causing physical limitations and decreased QoL [2]. Since IC mainly derives from atherosclerosis, patients with IC are at significant risk of cardiovascular morbidity [13]. The risk factors consist of smoking, hypertension, hyperlipidaemia, and diabetes mellitus; accordingly, secondary prevention with smoking cessation, physical activity, dietary changes, and best medical therapy (BMT, i.e., cholesterol reduction, antithrombotic drugs, and blood pressure control) are necessary for patients with IC to prevent progression of the atherosclerosis process and to prevent further cardiovascular morbidities [1]. Research has shown that there are differences between men and women with PAD. A systematic review showed in a meta-analysis that women tend to present more often with atypical leg symptoms compared to men and the review recommend that data on men and women should be reported separately [14]. This illuminates the importance of studying differences between men and women with IC in other variables as well.

Research has shown the importance of patients’ adherence to treatment in relation to QoL. Positive associations between adherence and QoL have been shown in other conditions, such as myocardial infarction, [15] diabetes mellitus, [16] and hypertension [17]. Besides the association between adherence to treatment and QoL, research in patients with coronary heart disease has reported positive associations between illness perceptions and QoL, where patients experiencing lower personal- and treatment control, lower illness coherence, or a cyclic timeline belief reported lower QoL [18]. However, research about associations between adherence to treatment, QoL, and illness perception in patients with IC is lacking.

Patients with PAD shape their own understandings of their conditions, which may influence their management of their disease and adherence to treatment [19]. The common-sense model (CSM) is a theoretical framework describing illness perception as a process for managing a health threat. The CSM includes perception, interpretation, and response to a health threat and consists of both cognitive and emotional representations in five subscales (identity, timeline, cause, control and cure, and consequences) [20]. Studies have shown associations between illness perception and adherence to secondary prevention in patients with coronary heart disease [21, 22].

Adherence to secondary prevention medication is vital to reduce the risk of morbidity and mortality [23, 24]. However, low adherence to medical treatment has been reported in patients with IC, where only two thirds are taking antiplatelets and statins five years after diagnosis [25, 26]. Adherence to long-term treatment has been associated with several influencing factors, including those related to the patients (such as illness perception and self-efficacy) [27]. Additionally, beliefs regarding both medicines and illness together with self-efficacy need to be addressed to better understand adherence to medication treatment [28]. Another important factor for adherence is health literacy, which is described as the ability to “access”, “understand”, “appraise”, and “apply” health-related information. Health literacy influences self-management behaviours and individual outcomes in chronic diseases. Patients with IC having inadequate or problematic health literacy reported both lower self-efficacy and worse QoL [29]. Self-efficacy is described as an individual’s belief in their own capacity to execute behaviours necessary to face challenges and to complete a specific task [30]. For instance, among patients with PAD, better self-efficacy was associated with better walking ability [31]. Importantly, individuals with limited self-efficacy tend to avoid setting goals and experience low confidence in their ability to succeed in the task ahead, [30] leading to lower QoL [32], but nurse-led self-management programmes can enhance patients’ self-efficacy [33].

There is evidence that illness perception, health literacy, self-efficacy, adherence to treatment and QoL influence the self-management in chronic diseases. Therefore, the contribution of the current study is to generate knowledge of these factors in patients with IC during the natural course of the disease the first year after diagnosis. This knowledge could further be used to improve strategies regarding secondary prevention care for patients with IC to increase their QoL. Accordingly, this study aimed to compare illness perception, health literacy, self-efficacy, adherence to treatment, and QoL in patients with intermittent claudication.

The analysis was based on the following research questions:

What do patients with IC believe cause their illness?

What differences are there in illness perception between men and women and between patients with sufficient and insufficient health literacy?

What associations are there between illness perception, adherence, self-efficacy and quality of life?

What are the predictors of quality of life?

What changes are there in self-efficacy and quality of life during the first year after diagnosis?

## Method

### Design

**Fig. 1:**
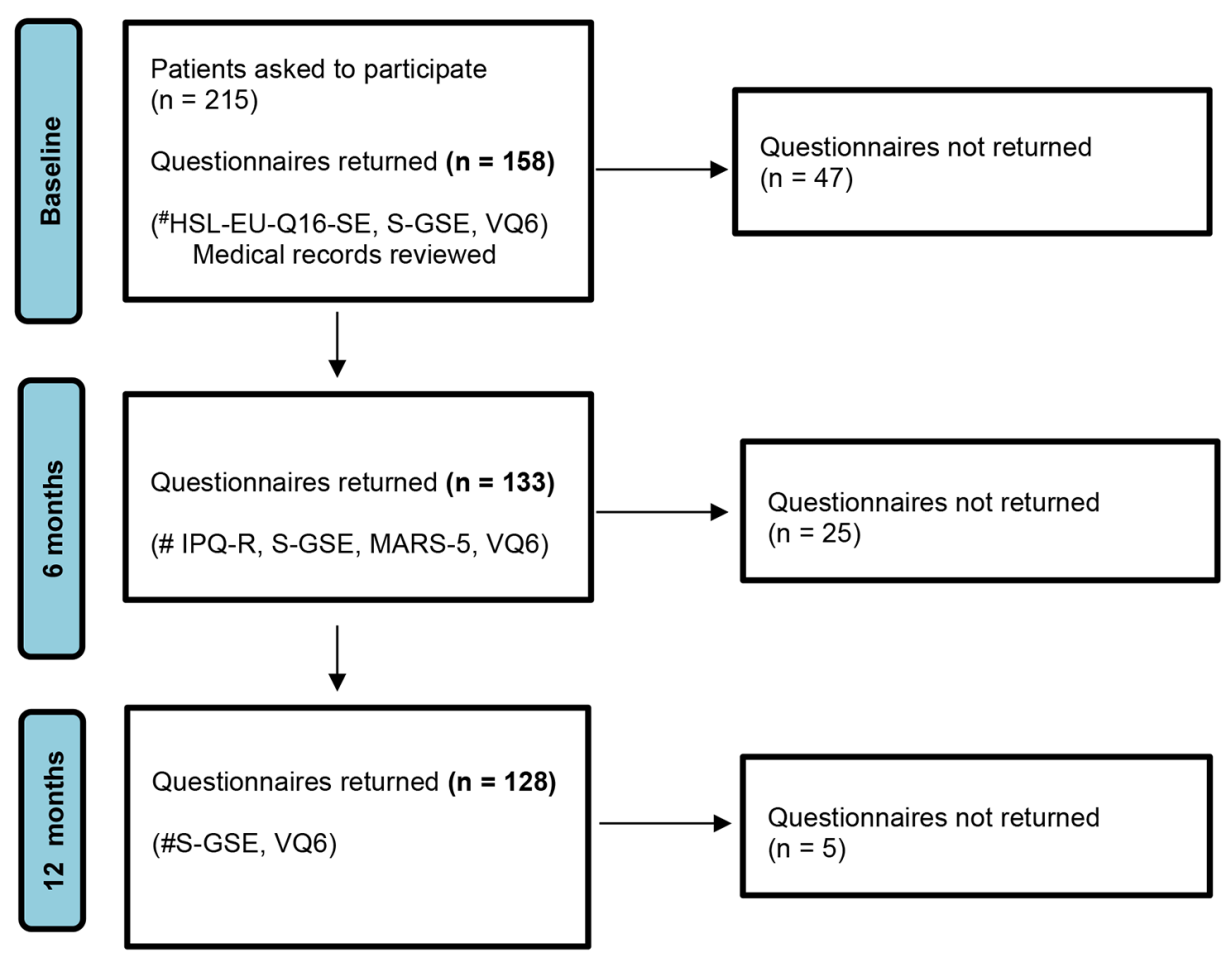
Flow chart of data collection. #Abbreviations for questionnaires: HSL-EU-Q16-SE: Health literacy S-GSE: Self-efficacy VQ6: Quality of life IPQ-R: Illness perception MARS-5: Adherence to treatment.

This was a longitudinal cohort study in which the participants filled in the questionnaires at three different occasions (baseline, 6 months, and 12 months), and data from medical records were extracted at baseline (Fig. 1). A consecutive sample method was used to select participants. This means that all patients visiting three outpatient clinics for vascular diseases located in southern Sweden meeting the inclusion criteria were invited to participate during the time of the study.

### Participants

Patients with a referral to one of the participating vascular clinics were recruited at their first visit to the outpatient clinic. At this visit, they were diagnosed with IC. All patients visiting these clinics received BMT, including medicine prescription and information of the importance of walking exercise and smoking cessation. The patients fulfilling the inclusion criteria were asked for participation by registered nurses specialized in vascular diseases. The inclusion criteria were patients diagnosed with IC (defined by clinical findings and ankle brachial index [ABI] < 0.9) and the ability to read and understand Swedish. Since the aim was to investigate the natural course of IC, patients with more severe PAD (rest pain, ulceration, and gangrene) or previously received surgical treatment for IC were excluded since their experience of the disease might differ from the patients newly diagnosed. The sampling procedure is accounted for in Fig. 1.

### Data collection

The data were collected between 2018 and 2020. Patients interested in participation received oral and written information during the referral visit. The information was given by registered nurses specialized in vascular diseases at the three clinics included in the study. Baseline questionnaires (background data, health literacy, self-efficacy and QoL) and informed consent forms were handed out during the referral visit with the advice to fill in the forms at home, thus giving the patients time to consider the decision to participate. One reminder was sent to non-responders. The questionnaires were sent back to the research team using prepaid envelopes. For the follow-up, the patients received new sets of questionnaires by mail after 6 months (illness perception, self-efficacy, adherence and QoL) and after 12 months (self-efficacy and QoL) together with prepaid envelopes. Two reminders were sent to non-responders. Background data − such as demographics, co-morbidities, clinical characteristics, and lifestyle factors − were collected through a self-reported questionnaire developed only for this study. The lifestyle factors physical activity and smoking were included based on the importance of secondary prevention for patients with PAD [1, 13]. Physical activity was self-reported, and the two questions were phrased as follows: “Are you being physically active?” (*yes/no*) and “How many times per week are you being physically active?” (*1*–*2*, *3*–*4*, *> 4*). ABI, blood pressure, cholesterol level, and blood glucose were collected from medical records at baseline. The data collection process is presented in Fig. 1.

### Questionnaires

Data on illness perception, health literacy, self-efficacy, adherence to medication treatment, and QoL were collected through self-administered questionnaires described below.

#### Illness perception

The Revised Illness Perception Questionnaire (IPQ-R) was based on the Common Sense Model of self-regulation [34] and revised with the following subscales: timeline acute/chronic, timeline cyclical, consequences, personal control, treatment control, illness coherence, and emotional representations, [35] which are accounted for in Table 1. The Swedish version of the instrument was validated and contains 38 items regarding perceptions of the illness and 18 items about causes of the illness. It uses a five-point Likert scale from *strongly disagree* to *strongly agree.* The last item has an open-ended response where the patient should list the three most important causal factors of the disease [36]. On subscales with 6 items a maximum of 2 missing items were allowed. Cronbach’s alphas for the subscales in the current study were α = 0.82 for timeline acute/chronic, α = 0.79 for timeline cyclical, α = 0.71 for consequences, α = 0.65 for personal control, α = 0.79 for treatment control, α = 0.92 for illness coherence, and α = 0.86 for emotional representations.

**Table 1:** Interpretation of IPQ-R subscales (Moss-Morris et al., 2002)

IPQ-R subscale	Score range	Definition	Interpretation of high scores
Timeline	6–30	Evaluates duration of illness	The patient believes the illness is enduring
Timeline cyclical	4–20	Evaluates views on cyclical nature of illness	The patient believes the illness is cyclical
Consequences	6–30	Evaluates views on negative consequences for patient	The illness has negative consequences on the patient’s life
Personal control	6–30	Evaluates views on the effect of personal control by the patient on the illness	The patient has high level of control over the illness
Treatment control	5–25	Evaluates views on the effectiveness of treatments available	The treatment is effective for the illness
Illness coherence	5–25	Evaluates the understanding of the illness	The patient has greater understanding of the illness
Emotional representations	6–30	Evaluates how the illness affects the patient emotionally	There is a greater emotional impact associated with the illness

#### Health literacy

European Health Literacy Survey Questionnaire 16 (HLS-EU-Q16-SE) was validated and translated to Swedish and is a short version of the European Health Literacy Survey Questionnaire (HLS-EU). It consists of 16 items in four different dimensions: “access/obtain”, “understand”, “process/appraise”, and “apply/use” health related information. It uses a four-point Likert scale: *very easy*, *easy*, *difficult*, *very difficult.* The responses *very easy* and *easy* were combined into one category (scored with one) and the responses *very difficult* and *difficult* were combined into another category (scored with zero). A sum score was calculated for respondents answering at least 14 questions [37] and divided into the three categories inadequate (0–8), problematic (9–12), and sufficient (13–16) [38]. The three health literacy levels were dichotomized into two levels: “insufficient” (“inadequate” and “problematic”*)* and “sufficient” health literacy [39]. Cronbach’s alpha was 0.91 in the current study.

#### Self-efficacy

The General Self-Efficacy Scale (S-GSE) is an instrument measuring the strength of an individual’s belief in their own ability to deal with difficult situations and setbacks [40]. The Swedish version of the instrument was validated [41, 42]. It consists of 10 items rated on a four-point Likert scale (*not all true* to *exactly true*). A total score was calculated where a high score indicates a high self-efficacy [40]. All the questions needed to be answered in order to be included in the analysis. Cronbach’s alpha was 0.92 in the current study.

#### Adherence to treatment

The Medication Adherence Report Scale (MARS-5) is the instrument used to determine patients’ self-reported adherence to medication treatment in general. In the current study, the patients were advised to report adherence specifically to the medication treatment for IC. The MARS consists of five items and uses a five-point Likert scale ranging from *always* to *never.* All questions needed to be answered in order to be included in the analysis. The instrument is valid and available in Swedish [43]. A total score was calculated where a higher score indicates a higher level for medication adherence [44]. Cronbach’s alpha was 0.57 in the current study, which is below the recommended value (α = 0.70) [45].

#### Quality of life

The Vascular Quality of Life Questionnaire-6 (VQ6) is a disease-specific instrument for the assessment of QoL in patients with PAD and is a short version of the Vascular Quality of Life Questionnaire (VascuQoL) [46]. The instrument was translated into Swedish and showed good psychometric properties [47, 48]. It consists of six items divided into five subscales: activity (two items), emotional, pain, social, and symptoms. The items are rated on a four-point Likert scale. The answers were summed to a total score (6–24), where a high score indicates a high quality of life [49]. All the questions needed to be answered in order to be included in the analysis. In the current study, Cronbach’s alpha was 0.88.

### Statistics

For analysing the data, IBM SPSS Statistics for Windows (Version 26.0. Armonk, NY: IBM Corp) was used. A p-value of 0.05 was set to determine the significance. For categorical variables, frequencies and percentages were calculated to describe the data, and the chi-square test was used for analysing differences in proportions. Normally distributed ordinal variables were presented as mean with standard deviation, and an independent samples t-test was used to compare groups. Non-normally distributed ordinal variables were presented as median with first and third interquartile ranges, and a Mann-Whitney U-test was used to analyse differences between groups. Normal distribution was assessed using histograms and the Shapiro-Wilk test. Spearman’s correlation analysis was conducted between the subscales of illness perception, self-efficacy, adherence to medication treatment, and quality of life. A linear multiple regression was conducted with the significant variables from the Spearman’s correlation analysis and with QoL as a dependent variable. For variables with repeated measures, the Wilcoxon signed-rank test was used to analyse differences, and the significant levels were adjusted with a manual Bonferroni correction. Missing data were handled according to pairwise deletion[50]. The questionnaires’ internal consistency was tested using Cronbach’s alpha coefficient.

## Results

A total of 158 patients were included in the study, of which 133 patients continued participation at 6 months and 128 continued at 12 months, resulting in a response rate of 73.5% at baseline, 61.9% at 6 months, and 59.5% at 12 months (Fig. 1). The study population at 6 months consisted of 66 men and 67 women, with a mean age of 75 years (age range 47–92). In the comparison between men and women, a difference was shown in civil status, where living alone was more common among women (p < 0.001). No differences appeared in age, ABI, education level, or lifestyle factors between men and women. The background characteristics of the study sample at baseline are presented in Table 2.

**Table 2:** Clinical characteristics, demographics, and lifestyle factors at baseline in subjects with intermittent claudication

	Total (n = 158)	Women (n = 80)	Men (n = 78)	p-value
Age (years)	74.1 (7.3)	75.0 (6.6)	73.3 (7.3)	0.160
Ankle brachial index	0.65 (0.19)	0.62 (0.19)	0.67 (0.19)	0.114
Body mass index (kg/m^2^)	25.8 (4.1)	24.8 (4.4)	26.8 (3.6)	**0.003**
Diastolic blood pressure (mmHg)	76.6 (10.5)	73.9 (9.1)	79.6 (11.3)	**0.006**
Systolic blood pressure (mmHg)	148.2 (21.2)	150.7 (21.3)	145.7 (20.9)	0.155
Cholesterol (mmol/l)	4.7 (1.3)	5.1 (1.3)	4.4 (1.3)	**0.012**
Blood glucose (mmol/l) *	6.2 (5.6–7.5)	6.1 (5.6–7.2)	6.4 (5.8–8.3)	0.811
*Civil status n (%)*				
Living alone	53 (33.5)	38 (47.5)	15 (19.2)	**0.001**
Cohabitation	89 (56.3)	37 (46.3)	52 (66.7)	**0.001**
Live-apart	5 (3.2)	3 (3.8)	2 (2.6)	0.752
*Education level n (%)*				
Elementary school	44 (27.8)	21 (26.3)	23 (29.5)	0.614
Upper-secondary school	45 (28.5)	25 (31.3)	20 (25.6)	0.465
Vocational school	26 (16.5)	10 (12.5)	16 (20.5)	0.163
University	42 (26.6)	24 (30.0)	18 (23.1)	0.349
*Lifestyle factors n (%)*				
Physical activity	125 (79.1)	65 (82.3)	60 (76.9)	0.495
Never smoker	16 (10.1)	9 (11.3)	7 (9.0)	0.655
Former smoker	108 (68.4)	50 (62.5)	58 (75.3)	0.083
Current smoker	33 (20.9)	21 (26.3)	12 (15.4)	0.101

Values are means (standard deviation) or number (percentage) of participants in each group.

*Median (Inter quartile range).

### Illness perceptions

The participants reported what they believed caused their illness, namely, IC. Of the 110 participants that answered the question regarding the causal factors, smoking was the most reported (42.7%), followed by age (21.8%), genetics (9.1%), and no idea (7.3%).

#### Comparison between men and women

In an illness perception comparison between men and women, women reported higher illness coherence, indicating greater understanding of IC than men (p = 0.028). Women also reported higher emotional representations associated with IC (p = 0.047) compared to men (Table 3).

#### Comparison between sufficient and insufficient health literacy

Patients with sufficient health literacy reported less consequences (p = 0.003) and lower emotional representations (p = 0.001) of the IC than patients with insufficient health literacy. They also reported higher self-efficacy (p = 0.049) and higher QoL (p = 0.005) than patients with insufficient health literacy (Table 3).

**Table 3:** Differences between men and women and health literacy regarding illness perception, self-efficacy, adherence to medication treatment, and quality of life at six months in subjects with intermittent claudication

Variables*	Total (n = 133)	Women (n = 67)	Men (n = 66)	Insufficient health literacy (n = 70)	Sufficient health literacy (n = 61)	p-value (w/m)*	p-value (insuff /suff )*
Illness perception subscales							
*Timeline* *missing (n = 11)*	24.0 (20.3–26.6)	24.0 (20.0–26.4)	24.0 (21.0–27.0)	23.5 (19.8–26.1)	24.0 (20.9–27.0)	0.752	0.309
*Timeline cyclical* *missing (n = 11)*	10.0 (8.0–13.0)	10.0 (8.0–13.0)	11.0 (8.0–13.0)	10.0 (8.0–12.8)	10.9 (8.0–14.0)	0.785	0.392
*Consequences* *missing (n = 7)*	17.5 (14.0–21.0)	18.0 (14.0–19.8)	17.0 (15.0–21.0)	19.0 (15.8–21.0)	16.5 (14.0–18.0)	0.432	**0.003**
*Personal control* *missing (n = 7)*	19.0 (17.0–22.0)	18.5 (17.0–22.0)	19.0 (17.3–21.8)	19.0 (17.0–21.0)	19.0 (17.8–22.0)	0.840	0.325
*Treatment control* *missing (n = 15)*	16.0 (13.8–18.0)	16.0 (14.0–18.0)	16.0 (13.0–18.0)	15.0 (13.4–18.0)	16.1 (14.0–18.0)	0.856	0.376
*Illness coherence* *missing (n = 13)*	19.0 (15.0–21.0)	20.0 (15.3–24.0)	17.0 (13.5–20.0)	18.0 (15.0–21.0)	19.0 (15.0–23.5)	**0.028**	0.194
*Emotional representations* *missing (n = 5)*	17.0 (13.0–20.8)	18.0 (13.0–22.0)	16.0 (13.0–18.0)	18.0 (14.0–22.3)	15.0 (12.3–18.0)	**0.047**	**0.001**
Self-efficacy *missing (n = 12)*	30.0 (26.5–33.5)	30.0 (26.0–34.0)	30.0 (27.0–32.0)	30.0 (26.0–32.0)	31.0 (28.0–35.0)	0.740	**0.049**
Adherence to treatment *missing (n = 6)*	25.0 (24.0–25.0)	25.0 (24.0–25.0)	25.0 (24.0–25.0)	25.0 (24.0–25.0)	25.0 (24.0–25.0)	0.496	0.223
Quality of life *missing (n = 5)*	15.0 (11.0–17.0)	15.0 (11.0–17.0)	16.0 (11.5–17.0)	13.0 (10.0–17.3)	16.0 (14.0–17.0)	0.152	**0.005**

*Median (IQR)

w: women, m: men, insuff: insufficient health literacy, suff: sufficient health literacy

#### Correlation

The subscales timeline (r_s_ = − 0.24; p = 0.009), timeline cyclic (r_s_ = − 0.19; p = 0.042), personal control (r_s_ = − 0.19; p = 0.044), and emotional representations (r_s_ = − 0.58; p < 0.001) correlated negatively with adherence to medication treatment, indicating that higher scores on these subscales were associated with lower adherence. The subscales treatment control (r_s_ = 0.22; p = 0.016) and illness coherence (r_s_ = 0.21; p = 0.028) correlated positively with adherence to medication treatment, indicating that higher scores on these subscales were associated with higher adherence (Table 4).

### Quality of life

Positive associations were found between personal control and QoL (p < 0.001) and between self-efficacy and QoL (p = 0.014), indicating that higher scores were positively associated with higher QoL. A negative association was found between the consequences of the illness and QoL (p < 0.001) as well as between adherence to medication treatment and QoL (p = 0.013), indicating that experiences of higher consequences of IC and higher adherence to medication treatment were associated with lower QoL (Table 4).

**Table 4:** Spearman’s correlation analysis between illness perception, self-efficacy, adherence to medication treatment and quality of life in subjects with intermittent claudication

Variables	Timeline	Timeline cyclic	Consequences	Personal control	Treatment control	Illness coherence	Emotional representations	Self- efficacy	Adherence to treatment
**Timeline cyclic**	−0.262 p-value **(0.004)**								
**Consequences**	0.190 p-value **(0.037)**	−0.116 p-value (0.204)							
**Personal control**	−0.171 p-value (0.062)	0.144 p-value (0.116)	−0.170 p-value (0.058)						
**Treatment control**	−0.283 p-value **(0.002)**	0.006 p-value (0.945)	−0.103 p-value (0.267)	0.469 p-value **(< 0.001)**					
**Illness coherence**	0.127 p-value (0.176)	−0.146 p-value (0.113)	−0.025 p-value (0.783)	0.322 p-value **(< 0.001)**	0.372 p-value				
**Emotional representations**	0.005 p-value (0.953)	0.103 p-value (0.260)	0.488 p-value **(< 0.001)**	−0.079 p-value (0.379)	−0.043 p-value (0.643)	−0.170 p-value (0.063)			
**Self- efficacy**	0.067 p-value (0.484)	0.085 p-value (0.368)	−0137 p-value (0.142)	0.074 p-value (0.432)	−0.081 p-value (0.394)	−0.130 p-value (0.165)	0.072 p-value (0.427)		
**Adherence to treatment**	−0.239 p-value **(0.009)**	−0.188 p-value **(0.042)**	−0.084 p-value (0.356)	−0.183 p-value **(0.044)**	0.224 p-value **(0.016)**	0.205 p-value **(0.028)**	−0.577 p-value **(< 0.001)**	−0.057 p-value (0.540)	
**Quality of life**	−0.175 p-value (0.057)	0.076 p-value (0.415)	−0.568 p-value **(< 0.001)**	0.348 p-value **(< 0.001)**	0.114 p-value (0.238)	−0.010 p-value (0.917)	−0.108 p-value (0.243)	0.224 **p-value (0.014)**	−0.224 **p-value (0.013)**

#### Factors related to QoL

A multiple regression model explained 46.5% of the variance in QoL (adjusted R square = 0.465; p < 0.001) and showed that both consequences and adherence to medication treatment were negative predictors of QoL, meaning that one unit increase in these variables decreased QoL (Table 5).

**Table 5:** Multiple linear regression at six months with quality of life as dependent variable

Independent variables	B	Standard error	β	p-value
Consequences in illness perception	−0.569	0.074	−0.576	**< 0.001**
Personal control in illness perception	0.167	0.087	0.143	0.057
Self-efficacy	0.096	0.056	0.123	0.088
Adherence to treatment	−0.556	0.196	−0.204	**0.050**

R square: 0.445

Adjusted R-square: 0.465

Sign: < 0.001

### Changes over time

The QoL medians with inter quartile range (IQR) were 13 at baseline (11–17), 15 at 6 months (11–17), and 16 at 12 months (12–18.8). A significant increase in QoL was seen between baseline and 6 months (p = 0.033), baseline and 12 months (p < 0.001), and 6 months and 12 months (p = 0.031). After a correction for multiple comparisons (Bonferroni), the significant increase in QoL remained between baseline and 12 months (p = 0.003). No significant changes over time were seen in self-efficacy.

## Discussion

The present study showed that illness perception differed in patients with IC in relation to sex and health literacy level. Patients’ self-reported beliefs on the causes of IC were smoking, age, and genetics. However, even if smoking was the most commonly reported causal beliefs for the disease (42%), the majority of the participants (89%) were current or former smokers. This is in line with a previous review on illness perception in patients with PAD, which showed that the patients had different beliefs regarding smoking as a causing factor for the disease; only some acknowledged smoking as a causing factor [19]. This has also been seen in patients with COPD, who expressed uncertainty about the connection between smoking and the disease [51]. The fact that 7.3% had no idea what caused their disease emphasizes a need to improve the patients’ awareness of the aetiology of the disease. As illness perception clearly influences patients’ beliefs about the causes of IC, an educational intervention addressing their perceptions could be one option to increase their awareness and, in turn, also their QoL. For example, previous research has shown that an illness perception correction-based educational programme provided via phone calls by a nurse improved QoL in patients with heart failure [52].

In a comparison between men and women concerning illness perception, women reported higher illness coherence and higher emotional representations, indicating they have greater understanding and greater emotional impact associated with IC compared to men. The difference in illness coherence contrasts with Al-Smadi et al.’s [53] review of illness perception in patients with coronary heart disease, where four studies [36, 54–56] reported no differences between men and women and two studies [57, 58] reported higher illness coherence in men than in women. The difference in emotional representation is in line with a review on sex differences in patients with chronic disease, where women experienced more negative stress about their condition compared to men [59]. Differences between men and women with PAD have also been studied earlier. A review showed that men and women differ in the presentation of PAD symptoms, where women more often present atypical symptoms and rest pain and, less often, IC symptoms [14]. Since differences between men and women with IC occur both in illness perception and symptom presentation, sex differences in IC patients should be further studied. Health care professionals’ awareness of potential sex differences could create preconditions for more individualized information, which could further improve the patients’ self-management of secondary prevention.

Patients with sufficient health literacy reported less consequences and lower emotional representations of the IC than patients with insufficient health literacy. This concurred with previous studies on patients with COPD and cardiovascular disease, which showed that those with insufficient health literacy also experienced more emotional representations [60, 61]. The current study also showed higher self-efficacy and higher QoL among patients with sufficient health literacy compared to those with insufficient health literacy, which is in line with previous research [62, 63]. These findings suggest that improving patients’ health literacy may improve their self-efficacy, which in turn may positively influence their QoL. Since health literacy can be improved by information and structured education, [64] patients with IC may benefit from a more active support from the health care system. Different methods – such as motivational interviewing, individual or group support, and in-person or web- or telephone-based support – have been beneficial for improving health literacy in patients with chronic diseases [65]. Further research is needed to assess these methods for improving health literacy in patients with IC. As previously suggested, digital formats may be more accessible for patients and perhaps more cost-effective, but they need both testing and evaluation among patients with IC.

Consequences and adherence to medication treatment were negative predictors of QoL in patients with IC. That the perceived consequences of the disease have an impact on daily life has earlier been confirmed in a review on patients with PAD; the patients experienced involuntary isolation and loss of independence due to their disease, which affected their self-image [19]. In this study, the association between increased adherence to medication treatment and decreased QoL is a surprising result in need of consideration. A possible explanation might be that pain and loss of independence have a negative impact on QoL in patients with IC [2]. Since BMT aims to prevent cardiovascular events and decrease the atherosclerotic progression rather than relieve IC symptoms, [1] high adherence to medications does not directly affect QoL. This negative association between adherence to treatment and QoL has also been reported earlier in a review on patients with COPD. According to Agh et al., [66] the treatment with inhaler therapy in COPD may affect the patient’s daily life due to perceived stigma when using inhalers in public, which could explain increased QoL with low adherence. However, this reason is not applicable to patients with IC since the treatment with BMT would not affect the patients’ daily life. Since adherence to taking antiplatelets and statins decreases after five years, [25, 26] future research with a longer perspective and, importantly, a combination of different methods to measure adherence would further illuminate associations between adherence and QoL in patient with IC. Another important aspect is that health care professionals should always remember to ask patients about their adherence behaviour since it is necessary information to enable evaluations of initiated treatment.

When examining changes over time, this study found a significant increase in QoL between baseline and 12 months despite no systematic secondary prevention support. This is an interesting finding that could be explained in a review on illness perception in patients with PAD: the patients experienced a process of adaptation, where they accepted their new situation and found other values in life [19]. This corresponds to the description of QoL as the discrepancy between an individual’s hopes and expectations and present experiences [67].

Even if the improvement in QoL was rather small, having knowledge of the reason for this increase despite no interactive care or support would be valuable in the process of creating a sustainable care for patients with IC. However, this was not possible with the current study design, so further research is needed, preferably with a qualitative approach that can describe the individual experiences. The fact that self-efficacy did not change over time was an expected result since managing chronic disease processes can be demanding and motivation can decrease over time [68]. Moreover, improvements in self-efficacy most likely require support to achieve, which Sol et al [33] have shown in patients with cardiovascular conditions. Even though QoL improved one year after visiting the outpatient clinic for vascular diseases, improving patients’ health literacy, self-efficacy, and illness perception is necessary to further improve the self-management of the disease. Most likely, patients with IC could benefit from better support to create more control and understanding in their process of managing the disease in order to maintain or increase their QoL. This could in turn be beneficial for secondary prevention, which is the first line of treatment. However, to be able to achieve this in clinical practice new strategies are needed. Positive experiences of nurse-led units in terms of increased awareness of their disease and increased motivation for initiating lifestyle changes has been shown among patients with IC [69]. This could be one option to improve patients’ self-management. Another way could be through self-management programmes, gaming, mobile applications, or social media [32]. However, future research is needed to evaluate these methods in relation to patients with IC.

### Strengths and limitations

A strength of this study is that only validated instruments were used, thereby minimizing measurement bias. Another strength is that data extracted from medical records were used to describe medical status. However, a potential weakness is that all data from the questionnaires were self- reported, which can be inaccurate due to social desirability or failure to recollect adherence behaviour [70]. A validated instrument for evaluating physical activity would be preferable in future research. Since many of the patients scored maximum points in the adherence questionnaire, the potential ceiling effect needs to be considered. This makes it impossible to distinguish differences in adherence behaviour. Similar skewness in adherence reports has been found in previous studies [71–73]. Studies have shown self-reported questionnaire to overestimate patients’ adherence in other chronic conditions [74, 75]. Nevertheless, despite the limitation, self-reported medication adherence measures can provide valuable and useful information [70]. Further research on adherence in patients with IC is recommended, preferably using different research methods, such as refill-of-prescriptions records from pharmacies in combination with self-reported data.

The longitudinal design of the study enabled us to determine patterns over time. However, this study required the participants to fill in questionnaires on three occasions over one year. During this time, some of the participants decided to withdraw further participation. A further limitation might be that no power calculation was performed. The period of the data collection proceeded for two years in order to recruit as many participants as possible and was planned to continue. However, due to the pandemic, the recruitment had to stop earlier since patients with IC were not a prioritized group for care during this time, meaning that they were not called to visit the open clinic. Significant differences were found despite a somewhat small sample size; however, differences that were not detected may exist [76]. The internal consistency of the scales, which was measured through Cronbach’s alpha or psychometric tests, was appropriate (apart from MARS and in line with previous research [45, 54, 66]), which is to be considered a strength. The dropout rate during follow-up was acceptable, and patients ceasing to participate may be considered as an unavoidable attrition [77].

## Conclusion

Illness perception differs in some aspects in relation to the level of health literacy as well as between men and women. Further, the level of health literacy seems to be of importance for patients’ self-efficacy and QoL. The current result suggest a need for new strategies to improve health literacy, illness perception, and self-efficacy over time – for example, by providing more tailored information regarding secondary prevention, preferably by using e-health tools, in order to strengthen self-management to further improve QoL in patients with IC.

## Data Availability

The dataset is available from the corresponding author on reasonable request.

## Abbreviations

ABI: Ankle brachial index
BMT: Best medical therapy
CSM: Common Sense Model
HLS-EU: European Health Literacy Survey Questionnaire
HLS-EU-Q16-SE: European Health Literacy Survey Questionnaire 16 Swedish
IC: Intermittent claudication
IPQ-R: The Revised Illness Perception Questionnaire
IQR: Inter quartile range
MARS-5: Medication Adherence Report Scale
PAD: Peripheral arterial disease
QoL: Quality of life
S-GSE: General Self-Efficacy Scale
VascuQoL: Vascular Quality of Life Questionnaire
VQ6: Vascular Quality of Life Questionnaire-6
